# Changes in saliva and serum analytes in domestic pigs and wild boar experimentally infected with African swine fever virus

**DOI:** 10.1186/s13567-025-01598-6

**Published:** 2025-11-06

**Authors:** Tessa Carrau, Alba Ortín-Bustillo, Alberto Muñoz-Prieto, Paul Deutschmann, Virginia Friedrichs, Martin Beer, José Joaquín Cerón, Sandra Blome, Lorena Franco-Martínez

**Affiliations:** 1 Institute of Diagnostic Virology, Friedrich-Loeffler-Institut, Suedufer 10, 17493 Greifswald–Insel Riems, Germany; 2 Interdisciplinary Laboratory of Clinical Analysis, Interlab-UMU, Regional Campus of International Excellence ‘Campus Mare Nostrum’, University of Murcia, 30100 Murcia, Spain; 3 Moorepark Animal and Grassland Research Center, Teagasc, Irish Agriculture and Food Development Authority, Cork, P61 C996 Ireland

**Keywords:** African swine fever, biomarkers, oral fluid, porcine, serum, saliva, sialochemistry

## Abstract

African swine fever (ASF) has reached an unprecedented global spread and threatens domestic and wild pigs on all continents. In domestic pigs and wild suids outside Africa, the disease is associated with signs of a viral hemorrhagic fever, the pathogenesis of which has not been fully elucidated to date. To address these knowledge gaps, the study of biomarkers of different physiopathological pathways that can be measured in saliva, can provide important insights and contribute to a more comprehensive understanding of ASF pathogenesis. In the present study, animal experiments were performed with experimentally ASF virus (ASFV/ “Prenzlau 22”) infected domestic pigs (DP) and wild boar (WB). For this, analyses of various biomarkers in saliva from DP and WB were conducted at three different time points of ASFV infection. For comparative purposes, serum biochemistry, qPCR values, and clinical parameters (i.e., clinical score and rectal body temperature) were also assessed. Biomarker analyses in the saliva of the infected DP showed significant increases in haptoglobin, S100A8/A9, S100A12, total protein, adenosine deaminase and LDH. In WB, α-amylase was increased in saliva at 7 days post-infection. In addition, changes in biomarkers of stress and inflammation were observed in the serum of DP and WB. Overall, in this report we demonstrate notable alterations in saliva and serum analytes of ASFV-infected DP and WB, reflecting physiopathological mechanisms such as activation of the stress, immune system, and inflammation. In future studies, the potential of these analytes as biomarkers of the disease and as a tool to evaluate the response of the host to the infection should be undertaken.

## Introduction

African swine fever (ASF) is a viral infection that affects domestic and wild pigs. The clinical signs of ASF can vary depending on the strain of the virus and the individual porcine immune response [1, 2]. Common clinical signs include high fever, loss of appetite, and lethargy. Infected pigs may exhibit erythema, particularly on the ears, abdomen, and lower limbs. They may also experience respiratory distress and coughing. Other clinical signs can include diarrhea, vomiting, and neurological signs such as incoordination and tremor [1]. To date, more than 6545 cases in German wild boar (WB) and 19 outbreaks in domestic pig (DP) farms have been officially notified [3]. Molecular epidemiology has enabled the potential identification of the sources of the DP farms outbreaks, highlighting that WB play an important role in the maintenance of the disease and pose a significant risk to the pork industry [4–6].

The study of biomarkers in biofluids allows the early diagnosis of pathological states and diseases, their progression, or response to treatments, among other utilities [7]. There is a wide range of biomarkers that proved their usefulness in porcine disease diagnosis and monitoring. For example, to assess stress response, biomarkers such as cortisol or salivary α-amylase (sAA) have been used [8], while adenosine deaminase (ADA) was employed to measure activation of the immune system, playing a role in the differentiation of T lymphocytes, and being increased in diseased animals [9]. Biomarkers associated with acute-phase responses, such as haptoglobin (Hp), pig major acute phase protein (ITIH4, Pig-Map), total proteins, and calgranulins such as S100A8/A9 (calprotectin) and S100A12, provide valuable indicators of inflammatory processes and immune system activation [10, 11]. Furthermore, redox status biomarkers, including the ferric reducing antioxidant power assay (FRAP), are known to change during conditions of oxidative stress and sepsis [12]. Additionally, enzymes like lactate dehydrogenase (LDH) and creatine kinase (CK) are useful for evaluating muscle and tissue damage [13].

Classically, biomarkers were mostly measured in blood derivates such as serum or plasma. However, in the last years, the study of biomarkers using minimally or non-invasive samples has gained increased attention. Among these non-invasive samples, the use of saliva as biological sample provides a series of advantages such as an easy, simple, well tolerated and painless sampling. This contrasts with blood collection in pigs, which is highly stressful for both the animal and the staff in charge [14, 15]. Thus, saliva can be sampled and analyzed on farm level, without the need of specialized personnel, allowing rapid and efficient disease control while preserving animal welfare [16]. As a non-invasive matrix, saliva has proven potential as a biofluid to evaluate the health status of animals since analytes can provide information about different pathophysiological states such as stress, immune responses, inflammation, sepsis, redox status, or tissue damage [13].

The objective of this work was to study the possible changes in salivary biomarkers in ASFV infected DP and WB. To this end, as proof-of-concept, we have analyzed a comprehensive profile of analytes including α-amylase, cortisol, oxytocin, Hp, ITIH4, total protein, S100A8/A9, S100A12, ADA, FRAP, LDH and CK. In addition, most of these biomarkers were also measured in serum, for comparative purposes. These analytes were correlated to the clinical score and gross pathological findings at the end of the experiment.

## Materials and methods

### Experimental design

Seven crossbred DP and seven WB aged roughly five months were experimentally infected by oronasal inoculation with an ASFV-containing spleen suspension with 10^4^ HAU_50_ per mL of a German ASFV isolate. Daily monitoring of clinical manifestations was conducted, and animals were euthanized upon reaching a humane endpoint, defined as the point at which an animal exhibits severe clinical signs or distress that indicate irreversible suffering, necessitating euthanasia to prevent further pain or discomfort. The animal experiment was approved by the competent authority (Landesamt für Landwirtschaft, Lebensmittelsicherheit und Fischerei (LALLF) Mecklenburg-Vorpommern) under reference number 7221.3–2-011/19.

Animals received a unique ear tag to enable definite identification of individuals. All animals were kept in the same stable, and divided into two units, both groups being able to interact through fencing.

### Cells and virus

The inoculum was prepared from pooled spleen samples derived from three DP of a previous trial using the “Prenzlau 2022” ASFV isolate that originated from the city Prenzlau in the German state of Brandenburg. This field-isolated ASFV strain belonging to genotype II was identified as ASFV lineage III according to Forth et al. [6]. Details on the preparation procedure and titration of the spleen suspension are described elsewhere [17].

### Animal trial

#### Clinical monitoring

During the animal trial, clinical parameters were monitored following a standardized clinical scoring system based on established protocols [18]. The evaluated parameters included liveliness, posture, gait, skin alterations, ocular irritations, breathing, feed intake, and defecation [18, 19]. Each parameter was scored from 0 (normal) to 3 (severe), according to the intensity of the clinical signs observed. The cumulative clinical score (CS) was calculated daily and used to assess disease progression and define humane endpoints. A humane endpoint was implemented when the CS reached ≥ 15 points or in cases where the attending veterinarian deemed the suffering unjustifiable based on clinical judgment, adapted from Kosowska et al. [19]. Rectal body temperature (RBT) was measured only in domestic pigs, as wild boar do not tolerate RBT measurement without immobilization.

#### Animal sample collection

Saliva and serum samples were obtained before inoculation, which was considered the day 0 post-inoculation (dpi), and on 2-, and 7-dpi from all DP and WB. Saliva samples were obtained using polypropylene sponges (Esponja Marina, La Griega E. Koronis, Madrid, Spain) as previously described [7]. For blood collection, DP were restraint with a snout rope and WB were laid down by placing them in dorsal recumbent position. Blood samples were collected from the animals via puncture of the cranial vena cava. A non-vacuum system was used, consisting of a needle connected to an adapter, manually inserted into blood collection tubes. This method allows for controlled blood flow and minimizes the risk of vein collapse or hemolysis, which can occur with vacuum-based systems. The Sarstedt Monovette^®^ system was used for sample collection. Tubes with appropriate anticoagulants (e.g., EDTA or heparin, depending on the analysis) were selected based on the intended laboratory assays. To adhere to veterinary hygiene, all samples were inactivated upon collection using Tergitol-type NP-40 (NP-40) at 0.5% (*v*/*v*) concentration as described by Franco-Martínez et al. [20]. All samples were stored at −80 °C and shipped to INTERLAB-UMU (University of Murcia, Spain) facilities for further analysis.

### Measurement of biomarkers

Biomarkers were selected to assess stress and wellness (cortisol, oxytocin, and α-amylase), inflammation (haptoglobin, pig major acute phase protein, total protein, S100A8/A9 and S100A12), immune system (adenosine deaminase), oxidative stress (ferric reducing antioxidant power assay), and general status (creatine kinase, and lactate dehydrogenase), as reviewed previously [7]. In brief, biomarkers were measured using different techniques:

Cortisol (ng/mL), oxytocin (pg/mL), haptoglobin (Hp, mg/L), and pig major acute phase protein (ITIH4, µg/L) were measured using immunologic methods. The first three used AlphaLISA assays (PerkinElmer, Inc., Hopkinton, MA, USA) read with a 96-well fluorometry plate reader (PerkinElmer, Inc., Hopkinton, MA, USA). Cortisol was quantified using an indirect competitive assay developed with a commercial monoclonal antibody against cortisol (MA1–16,703, Thermo Scientific, Rockford, Illinois, USA) [21]. Oxytocin was measured using a previously validated direct competitive assay [21]. Hp was measured through an in-house indirect assay using an anti-Hp antibody obtained from mice cell hybridoma [13]. ITIH4 was measured with a porcine species-specific commercially available ELISA kit (Porcine ITIH4, ElabSciences, Houston, TX, USA) previously validated for its use in saliva of pigs [11].

Adenosine deaminase (ADA, Adenosine Deaminase assay kit, Diazyme Laboratories, Poway, CA, USA, expressed as UI/L [22]), α-amylase (a-Amylase, OSR6182, Beckman Coulter Inc., Fullerton, CA, USA, UI/L [23]) haptoglobin in serum (Hp, Haptoglobin, Beckman Coulter Inc., CA, USA, g/L), total protein (TP, Protein in Urine and CSF, Spinreact, Barcelona, Spain, g/dL in serum and mg/dl in saliva), S100A8/A9 (BÜHLMANN fCal Turbo® assay BÜHLMANN, Laboratories AG, Switzerland, mg/L [24]), ferric reducing antioxidant power assay (FRAP, homemade reagents, mmol/L [25]), lactate dehydrogenase (LDH, Beckman Coulter Inc., IU/L), and creatine kinase (CK, Beckman Coulter Inc., IU/L) were measured in an automated biochemical analyzer (Olympus AU400, Olympus Diagnostica GmbH, Ennis, Ireland) using commercial assays which were previously validated for their use in porcine samples [24, 26, 27].

### Detection of ASFV genome

DNA was extracted as previously described [28]. The respective qPCR was performed according to the protocol using the accredited Virotype 2.0 assay and cycle threshold (Ct) was recorded on a C1000 thermal cycler with the CFX96 Real-Time System (Biorad). Ct values were calculated automatically by the analysis software included with the commercial PCR kit, following the manufacturer’s instructions.

#### Statistical study

Due to the limited sample size (*n* = 7), distributional assumptions were assessed through visual inspection of histograms and Q–Q plots, in addition to exploratory normality tests (D’Agostino and Pearson omnibus test). As the data did not follow a normal distribution, a logarithmic transformation [Ln(X + 1)] was applied. Potential outliers were identified and removed using the ROUT method. Data from days 0, 2, and 7 post-infection were then analyzed using one-way ANOVA with Holm-Sidak correction for multiple comparisons, while keeping in mind the constraints of the sample size. Pearson’s correlation coefficient was used to assess linear relationships between analytes and CS, RBT, and Ct values. Analyses were conducted using GraphPad Prism version 8 (GraphPad Software, Inc).

## Results

### Clinical signs and clinical scores

**Figure 1:**
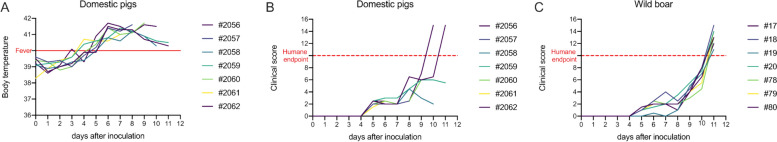
**The rectal temperatures of DP and clinical scores of DP and WB were taken each day after inoculation.** **A** Graphical summary of rectal temperatures after ASFV inoculation in DP. **B** and **C** Graphical summary of clinical scoring points of each DP and WB individual along the course of the trial.

Following inoculation of DP with “Prenzlau 2022”, pyretic onset occurred at 3-dpi (Figure 1A). From 5-dpi, pyrexia was accompanied by further clinical signs such as mild lethargy and decreased feed intake (Figure 1B). From then, the clinical scores of the pigs rapidly increased. Two DP reached the humane endpoint on 8- and 9-dpi for unjustifiable suffering presenting skin hyperemia, hematomas, and no feed intake. The remaining pigs reached the humane endpoint on day 10-dpi (1 animal) and 11-dpi (4 animals) with scores of 5.5, 9, 14, and 15 points (Figure 1B). All of these animals showed fever (mean 41.4 °C; Figure 1A), depression, anorexia, respiratory distress, conjunctivitis, skin hyperemia, hematomas, and deficiencies in the neuronal and musculoskeletal system. At this point, two pigs had developed hemorrhagic diarrhea, while one also presented bleeding from the nose.

Pyretic onset was not recorded for WB, but mild lethargy started in 6 animals at 5-dpi (Figure 1C). At 7-dpi, lethargy was accompanied by further clinical signs such as decreased feed intake and watery diarrhea in 3 WB. From then, the clinical scores of the pigs rapidly increased. All WB reached the humane endpoint on day 11-dpi with scores of 13, 9, 8, 7.5, 6.5 (2 animals) and 12 points (Figure 1C). All animals presented depression, anorexia, respiratory distress, conjunctivitis, skin hyperemia, hematomas, and deficiencies in the neuronal and musculoskeletal systems. At this point, one WB developed hemorrhagic diarrhea and seizures/convulsions.

**Figure 2:**
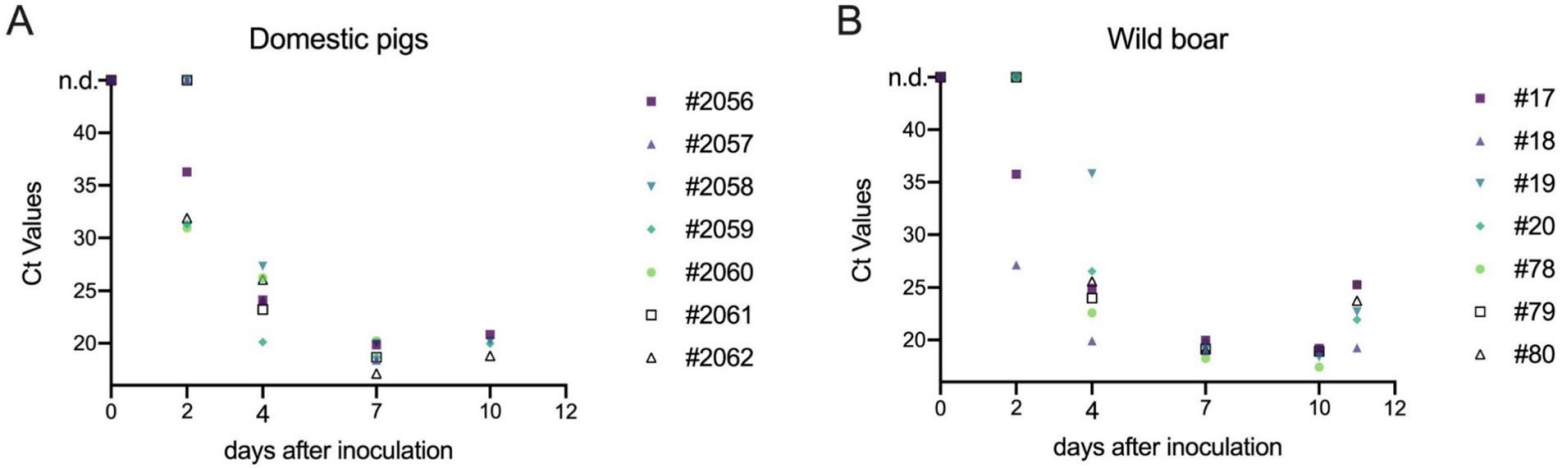
**Graphical summary of the cycle threshold values of DP (A) and WB (B) taken on 2, 4, 7, 10 and 11-days after inoculation with Prenzlau ASFV isolate.**

Blood samples of all animals were investigated by qPCR to detect the relative viral genome load in each individual. Irrespective of the animal group, Ct values were highly comparable between DP and WB and no significant differences could be found (Mann–Whitney test, *P* > 0.05). First detection of ASFV genome in blood was at 2-dpi in four DP and two WB. By 4-dpi, all animals were positive in the PCR (Figure 2). The lowest Ct values were reached on 7-dpi (Ct:17.12 in DP and Ct: 18.24 in WB; Figure 2B).

### Biomarkers in serum and saliva

Values of saliva and serum analytes obtained at 0-, 2-, and 7-dpi are shown in Tables 1 and 2 for DP and WB, respectively.

**Table 1:** **Biomarkers measured (median (25-75**^**th**^ **interquartile range)) in saliva and serum samples of DP before and after inoculation with ASFV.**

DPI	Amylase (UI/mL)	Oxytocin (pg/mL)	Cortisol (ng/mL)	Haptoglobin (mg/L. serum g/L)	ITIH4 (µg/L)	Total proteins (mg/dL, serum g/dL)	S100A8/A9 (mg/L)	S100A12 (µg/L)	ADA (UI/L)	FRAP (µmol/L)	LDH (UI/L)	CK (UI/L)
Saliva												
0	10.76 (0.99–10.91)	559 (533–857)	191.62 (57.51–403)	**1.26 (0.55–1.75)a**	38.63 (23.79–61.67)	**95.73 (68.59–143.36)a**	**0.12 (0.04–0.16)a**	**89.85 (58.03–130)a**	**835.2 (643.2–1164.8)a**	400 (297–850)	**176.8 (113.6–283.6)a**	
2	2.98 (1.91–8.04)	827 (668–985)	134.67 (124.3–145.08)	0.79 (0.69–1.11)	47.57 (29.41–78.77)	126.12 (115.61–147.02)	**0.01 (0.01–0.04)a**	37.04 (23.64–61.51)	**579.2 (326.4–1030.4)b**	1106 (726–1363)	**112 (71.6–189.2)b**	
7	8.34 (5.31–60.24)	731 (646–785)	55.24 (33.63–101.76)	**3.35 (2.28–8.98)a**	45.1 (37.95–77.75)	**151.91 (116.94–295.03)a**	**0.48 (0.39–0.5)a**	**987.52 (401.98–2655.35)a**	**7728 (5444.8–10,413.1)a,b**	462 (389–734)	**682.8 (489–1871)a,b**	
Serum												
0	**3.1 (2.88–3.93)a**			**0.52 (0.38–0.63)a**	**13.69 (12.14–37.85)a**	4.82 (4.77–5.04)	0.03 (0.03–0.04)	1.38 (1.21–1.61)	**5.6 (4.9–5.9)a**	349 (325–386)	**1276.6 (1235.4–1336.1)a**	**354.7 (294.9–418.8)a**
2	**2.9 (2.5–3.42)a**			**0.52 (0.3.–0.66)b**	**12.91 (11.56–31.39)b**	5.07 (4.71–5.14)	0.02 (0.01–0.02)	1.27 (0.77–1.65)	**5.1 (5–5.3)b**	361 (353–396)	**1200.5 (1056.9–1318.1)b**	**731.3 (504.2–1251.4)a**
7	**2.01 (1.96–2.76)a**			**3.27 (2.93–3.72)a,b**	**38.43 (32.37–148.07)a,b**	4.96 (4.32–5.1)	0.02 (0.01–0.05)	0.83 (0.51–1.01)	**14.9 (12.6–21.4)a,b**	380 (361–461)	**13148.8 (9740.8–28179.2)a,b**	**45312 (16537.6–102553.6)a**

ITIH4: pig major acute phase protein; ADA: adenosine deaminase; FRAP: ferric reducing antioxidant power assay; LDH: lactate dehydrogenase; CK: creatinine kinase.

Same letters and bold highlight differences of statistical significance (*P* < 0.05).

**Table 2:** **Biomarkers measured (median (25–75**^**th**^ **interquartile range)) in saliva and serum samples of WB before and after inoculation with ASFV.**

**DPI**	**Amylase (UI/mL)**	**Oxytocin (pg/mL)**	**Cortisol (ng/mL)**	**Haptoglobin (mg/L, serum g/L)**	**ITIH4** **(µg/L)**	**Total proteins (mg/dL, serum** **g/dL)**	**S100A8/A9 (mg/L)**	**S100A12 (µg/L)**	**ADA** **(UI/L)**	**FRAP (µmol/L)**	**LDH** **(UI/L)**	**CK** **(UI/L)**
Saliva												
0	**0.37 (0.12–0.65)a**	608.78 (545.79–954.29)	125.97 (63.36–168.77)	1.69 (1.29–2.16)	42.08 (28.19–49.43)	161.53 (97.36–291.53)	0.28 (0.2–0.6)	344.92 (260.85–830.3)	3660.8 (2614.4–6848)	530 (397–1334)	614.8 (572.8–1138)	
2	**0.57 (0.12–2.11)b**	691 (678.03–936.82)	152.18 (114.39–214.81)	2.8 (1.77–5.82)	28.37 (25.64–43.1)	220.44 (177.52–261.95)	0.36 (0.27–0.6)	255.85 (172.31–585.11)	2793.6 (1500.8–3809.6)	470 (382–588)	769 (372.1–1222.5)	
7	**22.38 (5–48.46) a,b**	946.2 (721.4–1066.98)	118.77 (70.56-192.03)	2.24 (1.36–3.77)	40.88 (31.05–48.45)	316.6 (140.12–448.52)	0.66 (0.33–0.81)	1447.54 (716.1–2139.78)	6960 (4334.1–8668.75)	802 (576–1143)	1256.2 (426.6–1579.3)	
Serum												
0	2.34 (2.23–2.87)			**0.78 (0.5.–1.48.)a**	**11.94 (10.98–15.82)a**	6.2 (6–6.38)	0.01 (0.01–0.02)	0.53 (0.07–0.69)	**7.1 (6.5–8)a**	434 (408–481)	**1149 (1033.5–1198)a**	316.9 (315.8–634.5)
2	2.18 (1.911–2.49)			1.58. (0.75.–3.77.)	14.365 (12.04–24.66)	6.58 (6.35–7.58)	0.04 (0.03–0.12)	3.42 (0.32–11.15)	10.8 (7.6–22.4)	646 (431–995)	**1346.4 (1327–3535.2)b**	668.3 (434.05–985.48)
7	1.9 (1.8–2.27)			**2.29 (1.52.–3.03.)a**	**27.49 (17.57–29.24)a**	6.55 (5.15–7.16)	0.03 (0.02–0.12)	0.42 (0.1–5.77)	**37.9 (28–45.6)a**	615 (474–800)	**8078.4 (6680–12131.2)a,b**	1929.2 (884–2704)

ITIH4: pig major acute phase protein; ADA: adenosine deaminase; FRAP: ferric reducing antioxidant power assay; LDH: lactate dehydrogenase; CK: creatinine kinase.

Same letters and bold highlight differences of statistical significance (*P* < 0.05).

In DP, saliva showed statistically significant increases in haptoglobin (2.66-fold), total protein (1.58-fold), S100A8/A9 (4-fold), S100A12 (11-fold), ADA (9.25-fold), and LDH (3.86-fold) at 7-dpi in comparison to 0-dpi. In addition, at 7-dpi, ADA and LDH were 13.34- and 6.1-fold higher than at 2-dpi. In serum, increases of statistical relevance in 7-dpi in comparison to 0- and 2-dpi were found for haptoglobin (6.28- and 6.22-fold higher), ITIH4 (2.8- and 2.98-fold, respectively), ADA (2.66- and 2.92-fold, respectively), LDH (10.3- and 10.95-fold, respectively). Serum amylase decreased and CK increased at 2- and 7-dpi in comparison to 0-dpi (1.06- and 1.54-fold lower for amylase, and 2.06- and 127.75-fold higher for CK, respectively).

**Figure 3:**
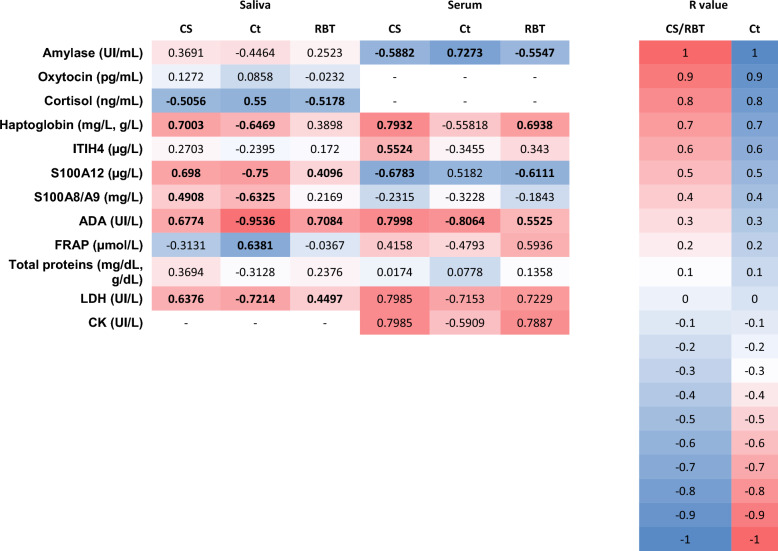
**Correlation matrix between measured biomarkers in DP and recorded clinical signs (CS and RBT) and relative qPCR values (Ct) depicted as a heatmap.** Heatmap represents the color-coded correlation factors. The color value of the cells is proportional to the strength of the associations, ranging from red (positive correlations for CS and RBT and negative correlation for Ct values) to blue (negative correlations for CS and RBT and positive correlation for Ct values). ITIH4: pig major acute phase protein; ADA: adenosine deaminase; FRAP: ferric reducing antioxidant power assay; LDH: lactate dehydrogenase; CK: creatinine kinase. Bold highlights differences of statistical significance (*P* < 0.05).

When correlations between biomarkers with CS, Ct and RBT were analyzed, correlations of statistical relevance were found for 17 salivary and 18 serum analytes (Figure 3). Of those, some strong (0.7 ≤ R value ≤ -0.7) correlations were found. CS strongly correlated with Hp in saliva, and with Hp, ADA, LDH and CK in serum; Ct showed strong correlations with S100A12, ADA, and LDH in saliva, and with amylase, ADA, and LDH in serum. Finally, RBT was strongly correlated with ADA in saliva, and with LDH and CK in serum.

In WB, salivary α-amylase was statistically significantly higher at 7-dpi in comparison to pre-infection measurements (60.48-fold) and 2-dpi (39.26-fold). In the serum, haptoglobin (2.92-fold), ITIH4 (2.3-fold), ADA (5.34-fold), and LDH (7.03-fold) were higher at 7-dpi when compared to 0 dpi; and LDH levels at 7-dpi were sixfold higher than those observed at 2-dpi.

**Figure 4:**
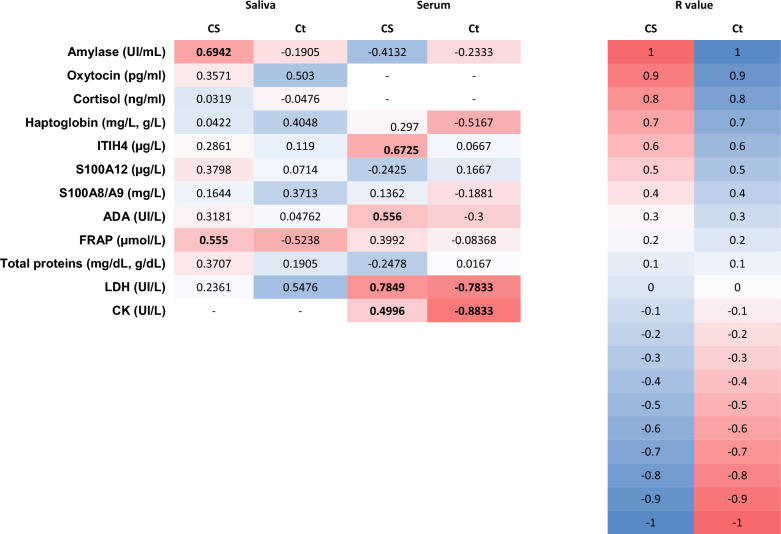
**Correlation matrix between measured biomarkers in WB and recorded clinical signs (CS) and relative qPCR values (Ct) depicted as a heatmap.** Heatmap represents the color-coded correlation factors. The color value of the cells is proportional to the strength of the associations, ranging from red (positive correlations for CS and negative correlation for Ct values) to blue (negative correlations for CS and positive correlation for Ct values). ITIH4: pig major acute phase protein; ADA: adenosine deaminase; FRAP: ferric reducing antioxidant power assay; LDH: lactate dehydrogenase; CK: creatinine kinase. Bold highlights differences of statistical significance (*P* < 0.05).

When correlations between biomarkers with CS and Ct were analyzed in WB, correlations of statistical relevance were found for 2 salivary and 6 serum analytes (Figure 4). Of those, in serum, LDH strongly correlated with CS and Ct, and CK in serum strongly correlated with Ct.

## Discussion

This study aimed to evaluate potential changes in serum and saliva of DP and WB following infection with ASFV, with the goal of advancing current knowledge on ASFV pathology. To this end, a panel of biomarkers previously validated in pigs was employed. Specifically, acute phase proteins such as Hp and ITIH4 and total proteins were used to assess the magnitude of inflammation [7], while calprotectins S100A8/A9 and S100A12 provided information on immune system activity [29]. ADA was also included as an immune marker, particularly indicative of lymphocyte activity [30]. Additionally, cortisol, α-amylase, and oxytocin were used to evaluate stress and welfare, oxidative stress was assessed via the FRAP assay, and tissue damage was evaluated using LDH and CK [7]. Therefore, the analysis of this panel of biomarkers may contribute to a more comprehensive understanding of the multifactorial physiological changes occurring during ASFV infection.

In our study, following an incubation period of four days, the clinical signs appear and all DP and WB exhibited an acute lethal disease course characterized by high fever, general depression, anorexia, ataxia, hemorrhages, hematomas, and respiratory distress; being consistent with earlier findings using highly virulent strains [31]. By 11-dpi, all animals had reached the humane endpoint or had succumbed acutely. It is noteworthy that the clinical signs observed were severe yet non-specific, leading to numerous differential diagnoses that could be of relevance in the field. Viral genome was detected in organs, blood, and serum of all animals, irrespective of the disease course (data not shown), consistent with prior studies [32]. Overall, these findings indicated the suitability of the model used in our report to study the acute course of the ASF infection.

Saliva sampling is a non-invasive method and less stressant and painful when compared to blood [7], especially in pigs where saliva collection is very well tolerated whereas blood sampling requires immobilization. Sialochemistry refers to the measurement in the saliva of a profile of analytes including those that are usually in serum biochemistry profiles. Sialochemistry in animals, especially in WB, is still very new. In this report, we have selected a profile of analytes that can cover the main physiopathological pathways that saliva can provide information about such as stress, inflammation, sepsis, redox and tissue homeostasis [7, 10]. In concrete, for this study, eleven biomarkers were measured in saliva, and 10 in serum samples, providing a general overview of the health status of the animals at two different times after ASFV infection.

Stress was assessed by α-amylase, oxytocin, and cortisol. α-Amylase is a biomarker of the autonomous nervous system activation and has been shown to increase with high-stress levels and long pain duration [33]. In our study, α-amylase was increased in saliva of wild boars 60-fold at 7-dpi. This enzyme has been shown to be highly increased (>1000 IU/L) when pigs are restrained [34, 35]. In our study, for DP, no changes of statistical relevance were observed for α-amylase in saliva, although it decreased at 2-dpi and at 7-dpi almost reached pre-infectious values. In serum, amylase gradually decreased, although it was only statistically significant in DP. Further research is necessary to understand the mechanisms behind the observed decrease in α-amylase and to clarify potential differences in stress physiology between wild and domestic suids. Oxytocin is considered a biomarker of positive emotions and social well-being in domestic animals [21]. In contrast to other stress biomarkers, oxytocin decreases following stress and pain. Recent studies have demonstrated reductions in salivary oxytocin concentrations four hours after transport [21]. Cortisol levels, on the other hand, rise with activation of the hypothalamic–pituitary–adrenal axis [36]**,** increasing in pigs under stress [33, 37]. In our study, oxytocin and cortisol were only measured in saliva in these samples because of the lack of a validated method for the measurement of oxytocin in serum, and due the insufficient serum sample volume for cortisol, and no statistically significant differences were observed in salivary oxytocin or cortisol levels. These findings suggest that the specific stress response triggered by ASFV infection may differ from those reported in other infectious diseases, where changes in these biomarkers have been more pronounced. Further research is needed to explore the underlying reasons for the lack of measurable changes in salivary oxytocin and cortisol in this context, and to determine whether different infection stages or sample matrices could reveal more consistent biomarker patterns.

Inflammatory and immune reactions caused during ASFV infection were evaluated through Hp, ITIH4, total protein, S100A8/A9 and S100A12. All of these biomarkers are validated in human and veterinary medicine in a variety of contexts including hemorrhagic diseases [38, 39], inflammation and sepsis [40, 41]. Although these biomarkers of inflammation are usually measured in serum, recent studies have proved their usefulness in saliva also. Hp can be measured in pigs’ saliva [42] being very sensitive for early detection of inflammation and can be increased even before clinical signs appear [7]. Salivary levels for ITIH4 (Pig major acute phase protein, Pig-Map) [11] and S100A8/A9 and S100A12 after stress, sepsis, and non-septic inflammation have been measured recently [24, 43]. However, divergences in responses in these analytes between both fluids are common [29, 43], and therefore, cautions had to be taken when comparing results between the two matrices. Overall, in our study, inflammatory reactions are present in both groups of animals after inoculation with ASFV. This was evident by increases in saliva at 7-dpi found for Hp, total protein, S100A8/A9, and S100A12 in DP. In serum, there were also increases in Hp and ITIH4 at 7-dpi in both species. Therefore, for DP, saliva was a suitable biofluid to detect inflammatory reactions, being more sensitive than serum to detect changes in S100A8/A9 and S100A12. This is in line with a recent study that showed increases in salivary S100A12 levels in pigs after LPS administration and turpentine administration, whereas no significant changes in serum were observed [43].

ADA, an enzyme ubiquitously expressed, plays a role in the differentiation of T lymphocytes and is increased in diseased animals [9, 44]. In humans, this enzyme has been evaluated as a biomarker of cell-mediated immunity and chronic inflammation, and previous works have shown an ADA increase after acute LPS challenge in pigs [8, 44]. In pigs, ADA in saliva is also increased in situations of lameness and prolapses and positively correlated with pain score [33, 45]. In our study, ADA levels showed a similar pattern in both biofluids of DP and WB saliva, with a decrease at 2-dpi followed by an increase at 7-dpi; while in serum of wild boars ADA gradually increased. The increases in ADA could be indicative of inflammation and activation of T lymphocytes typically caused by ASFV infection [46]. However, further studies evaluating both humoral and cell-mediated immune responses are needed to confirm this hypothesis.

Regarding oxidative stress, in our study, no changes of statistical relevance were observed in FRAP. FRAP was selected in this study because it is an economical, automated, and easy to perform assay that showed previously increases in saliva in other conditions in pigs [12]. However, it would be of high interest in future studies to evaluate other additional biomarkers of oxidative stress such as the cupric reducing antioxidant capacity (CUPRAC), trolox equivalent antioxidant capacity (TEAC), advanced oxidation protein products (AOPP), ferrous oxidation-xylenol orange (FOX), peroxide activity (POX-Act), or reactive oxygen-derived compounds (d-ROMs). These biomarkers showed changes in different diseases [12, 47] and would be interesting to assess if any of them could be more sensitive to detect changes in oxidative status in these conditions.

When tissues containing LDH such as musculoskeletal, heart or hepatocytes are hampered, it is released into the bloodstream. LDH is also increased during pulmonary or hematological disorders [48, 49], and in situations of reduced welfare in pigs, such as lameness or after nose snaring [34]. In our study, serum LDH levels at 7-dpi were 10- and 7-fold higher in DP and WB, respectively. In addition, increases in LDH were found in saliva one week after the infection. Similarly, since CK is mainly found in the cytoplasm of muscle cells when muscular tissues are damaged, it is released into the bloodstream and found increased in serum [50]. In these situations, CK has been also found higher in saliva [51]. In our report, serum CK increased 127-fold in DP. In wild boars, the increase was 6-fold but not statistically relevant, probably due to the inter-individual variability. Unfortunately, our method to measure CK in saliva required a high sample volume (50 µL) and, therefore, it could not be assessed. The changes observed in LDH and CK support the reduced welfare observed in these animals, as well as damage in different tissues that promoted the release of these biomarkers into the bloodstream.

When comparing saliva and serum as sources of biomarker information, we have observed some differences among animals. In DP, serum appears to be less sensitive than saliva in detecting changes in certain biomarkers, such as ADA, S100A8/A9 or S100A12, as previously reported elsewhere [24, 43, 45]. On the other hand, in WB, serum seems to be a more accurate biofluid for biomarker analysis, as it showed changes in more analytes when comparing pre and post-infection measurements. It is possible that for WB, implementing a different saliva sampling procedure that could reduce the manipulation and stress of the animal in the future might yield better results. As biomarker research advances, optimizing sampling techniques in different animal species will be crucial in obtaining reliable and comprehensive data for health assessment and disease monitoring.

Biomarkers were also evaluated in relation to the clinical parameters observed. Elevated RBT, commonly known as fever, is frequently associated with infection and inflammation, representing one of the most well-known clinical signs of ASFV infection [1]. Our study showed that in DP, RBT was strongly correlated with ADA in saliva, probably reflecting a status of activation of T lymphocytes, and with LDH and CK in serum indicating tissular damage. Clinical score in DP was correlated with Hp in saliva, and with Hp, ADA, LDH and CK in serum, indicating a correlation of the clinical score with markers of inflammation, immune system, and muscle damage. Ct values in DP were correlated with biomarkers of immune response, such as S100A12 and ADA in saliva, and tissue damage such as LDH saliva and serum in DP.

Overall, in DP, the measurement of analytes in saliva allowed to identify an inflammatory status, as well as activation of the cellular immune system and a situation of tissue damage. In WB, in the conditions of this trial, serum seemed more sensitive to detect changes in analytes. Correlations among clinical score, RBT, and Ct values were found with some of the biomarkers mentioned herein, probing their potential usefulness as health indicators in both species. This study also reinforced the utility of biomarkers for understanding and managing animal health, especially in scenarios involving complex diseases and novel research models.

The present study has some limitations. First, in agreement with the 3Rs principle (Replacement, Reduction, Refinement) and current ethical guidelines for animal experimentation, the number of animals used was minimized to the lowest possible while still ensuring scientifically meaningful results. For this reason, the inclusion of additional control animals solely for reference purposes was avoided, and pre-infection samples served as internal control. Therefore, the results of this study should be considered preliminary and further studies in a larger population and also in field conditions would be recommended for gaining more knowledge about the potential of saliva analysis in this disease. Second, although co-infections were not specifically ruled out by PCR or serological testing, precautions were taken to minimize this risk. All animals used in the study were clinically healthy and sourced from high-health-status breeding facilities. Upon arrival, they underwent an adaptation period of more than seven days, during which they were closely monitored for any clinical signs of disease or co-infection, none of which were observed. To prevent stress associated with handling and immobilization, RBT was not recorded in WB, which limited consistent monitoring across species. Last, the increase of the analytes in saliva could be influenced by the dehydration of the animals, and further studies should be performed to elucidate if different degrees of dehydration could influence salivary biomarkers.

Saliva and serum showed changes in selected analytes related to stress, inflammation, immune system and tissue damage after an experimental infection with ASFV. These changes were of different magnitude in saliva and serum and between domestic pigs compared to wild boards. Further studies should be performed to determine the potential use of these analytes as biomarkers of this disease.

## Data Availability

Raw data are available upon reasonable request to the corresponding author.
